# Urbanization level and medical adverse event deaths among US hospital inpatients over the period 2010–2019

**DOI:** 10.1016/j.pmedr.2022.101888

**Published:** 2022-07-05

**Authors:** Petteri Oura

**Affiliations:** a Department of Forensic Medicine, Faculty of Medicine, University of Helsinki, PO Box 21, FI-00014 Helsinki, Finland; b Forensic Medicine Unit, Finnish Institute for Health and Welfare, PO Box 30, FI-00271 Helsinki, Finland

**Keywords:** Urbanization, Adverse event, Epidemiology, Mortality, US

## Abstract

Urban-rural disparity constitutes a major source of health inequity also in high-income countries. This study aimed to compare the distribution of deaths due to medical adverse events across urbanization levels among US hospital inpatients. An open dataset from the National Center for Health Statistics (NCHS) comprised all certified deaths of US inpatients over the period 2010–2019. The urbanization level of each decedent was determined in accordance with the 2013 NCHS Urban-Rural Classification Scheme (large metropolitan, medium or small metropolitan, or nonmetropolitan). The outcome was death due to a medical adverse event (ICD-10 codes Y40—Y84) proportional to total inpatient deaths. The data were standardized for sex, ethnicity, and age, and analyzed with linear mixed models. Of the 8 071 907 certified inpatient deaths during the study period, 21 444 (0.27%) were primarily attributed to medical adverse events. Decedents who resided in medium or small metropolitans and nonmetropolitans had approximately 0.5 units higher rate of adverse events per 1000 deaths (corresponding to a relative differece of 20%) when compared to decedents who resided in large metropolitans. Moreover, the urban–rural gradients showed an increasing trend towards the end of the study period, as the difference was found to increase at a rate of approximately 0.1 units per year (3%). There were no statistically significant differences between decedents from medium or small metropolitans and nonmetropolitans. The present findings highlight gradients in adverse event deaths between geographic areas, providing a basis for targeted preventive efforts. Future studies are invited to elucidate the underlying phenomena.

## Highlights

• Urban-rural disparity is a major source of health inequity.
• Urban-rural gradients in adverse event deaths were explored among US inpatients.
• Decedents who resided outside large metropolitans had more adverse event deaths.
• The data highlight gradients in adverse event deaths between geographic areas.

## 1 Introduction

Although patient safety is recognized as a key health priority (Flott et al., 2019, National Academies of Sciences, Engineering, and Medicine et al., 2018, World Health Organization, 2021), the burden of harm caused by medical care remains high globally and in the US (Bates and Singh, 2018, Kruk et al., 2018, Slawomirski et al., 2017; The Lancet, 2019). In this study, the term “medical adverse event” is used for incidents of unintentional harm caused by medical care (Garrouste-Orgeas et al., 2012, Grober and Bohnen, 2005). Of patients and clients receiving health care services, inpatients are particularly vulnerable to non-fatal and fatal adverse events (Classen et al., 2011, Makary and Daniel, 2016, Schwendimann et al., 2018). It is thus imperative that further resources will be dedicated to the research and prevention of adverse events (Bates and Singh, 2018, Oura, 2021), starting with inpatient care.

Social well-being and equitable health are dependent on a wide span of societal factors referred to as the social determinants of health (Commission on Social Determinants of Health, 2008, NEJM Catalyst, 2017). As yet, social gradients in health and illness persist in both developing and developed countries (Commission on Social Determinants of Health, 2008). Given that a notable share of populations reside in rural areas (e.g., ca. 37% in the US) (Brezzi et al., 2011), urban–rural disparity constitutes a major source of health inequity also in high-income countries (Commission on Social Determinants of Health, 2008, Gong et al., 2019). Health inequity is driven by such factors as access to healthcare, availability of health care services, and socioeconomic wealth, all of which generally favor urban areas (Commission on Social Determinants of Health, 2008, Gong et al., 2019). As ethnic and socioeconomic gradients have been reported also in the patient safety context (Burstin et al., 1992, de Jager et al., 2020, Shen et al., 2016, Stockwell et al., 2019), the subsequent question is whether there are urban–rural gradients in non-fatal and fatal medical adverse events.

Understanding if and how urbanization level relates to an inpatient’s risk of sustaining medical adverse events will help highlight geographic areas at highest risk and direct preventive efforts accordingly. However, there currently is a paucity of up-to-date information regarding this aspect. A recent cross-sectional study from New Zealand addressed hospital harm among urban and rural patients, and found no evidence that rurality was associated with increased rates of harm (Atmore et al., 2021); in older US reports, adverse events appeared less likely in rural than in urban hospitals (Coburn et al., 2004, Vartak et al., 2010). As such, the presence and nature of potential urban–rural gradients in adverse events remain ambiguous. Moreover, few studies have specifically addressed fatal adverse events in the US, analyzing national data over a longer period of time.

This retrospective register-based study exploited national US cause-of-death data from 2010 to 2019, with the aim to compare the distribution of deaths due to medical adverse events across urbanization levels among hospital inpatients by means of a proportional mortality analysis. As the rural areas of US are generally characterized by lower socioeconomic position, lack of health insurance, and physician shortages (Gong et al., 2019), deaths attributed to adverse events were hypothesized to be more common among decedents who were residing in the rural parts of the country.

## 2 Material and methods

### 2.1 Database

The study exploited national publicly available data from National Center for Health Statistics (NCHS), Centers for Disease Control and Prevention (CDC), Department of Health and Human Services, US. The “Underlying Cause of Death” dataset was accessed in the CDC Wonder database (US Centers for Disease Control and Prevention, 2021) on May 21, 2022. The dataset was comprised of certified deaths of US residents, with causes of death communicated according to the 10th revision of the International Classification of Diseases (ICD-10) coding system. The study was based on publicly available anonymized databases and thus exempt from ethical approval.

To study fatal adverse events among hospital inpatients, search queries were delimited to adverse events as the primary (i.e., underlying) cause of death, and to decedents who were inpatients at the time of death. In order to account for secular trends in overall inpatient mortality, the total number of annual inpatient deaths was also collected.

### 2.2 Medical adverse event deaths

Deaths due to medical adverse events were identified by ICD-10 codes Y40—Y84 (“all adverse events”). This approach covered complications of medical or surgical procedures (Y83—Y84; “procedure-related events”), medication-related adverse events (Y40—Y59), medical or surgical misadventures (Y60—Y69), and device-related adverse events (Y70—Y82). However, due to low numbers of most adverse event subtypes in the data, the analysis focused on all adverse events in a pooled manner. NCHS data use restrictions do not permit publishing derivatives of death counts of nine or fewer.

### 2.3 Urbanization level

On the basis of the decedent’s legal residence, each death was assigned to an urbanization category according to the 2013 NCHS Urban-Rural Classification Scheme for Counties. The classification scheme is described in detail elsewhere (US National Center for Health Statistics, 2021). In brief, the classification operates at the county level and includes a total of six initial categories, of which four are metropolitan (i.e., Large central metropolitan, Large fringe metropolitan, Medium metropolitan, Small metropolitan) and two nonmetropolitan (i.e., Micropolitan, Noncore). The division between categories is based on the Office of Management and Budget’s 2013 definition of metropolitan statistical areas and micropolitan statistical areas, as well as US 2012 population estimates.

For this study, the three following categories were formed:

1) Large metropolitan (includes Large central metropolitan and Large fringe metropolitan): Counties in metropolitan statistical areas with a population of ≥ 1 million.
2) Medium or small metropolitan (includes Medium metropolitan and Small metropolitan): Counties in metropolitan statistical areas with a population of < 1 million.
3) Nonmetropolitan (includes Micropolitan and Noncore): Counties in micropolitan statistical areas, and counties that did not qualify as micropolitan.

The above categories were formed in order to perform meaningful comparisons between three clearly defined urbanization levels.

### 2.4 Other demographic data

To facilitate standardization of proportional mortality, basic data on sex, ethnicity, and age were collected from the annual mortality data. Adverse event deaths and total inpatient deaths were recorded in a piecewice manner for sex (male/female), ethnicity (white/other), and age strata (0–29/ 30–59/ 60+ years). The strata were relatively broad due to NCHS data use restrictions. The 2010 distributions were used as reference in the standardization procedure.

### 2.5 Statistical analysis

Stata/MP version 17 (StataCorp, College Station, TX) and IBM SPSS Statistics version 27 (IBM, Armonk, NY) were used to analyze the data. Microsoft Excel version 2005 (Redmond, WA) was used to draw mortality plots. The level of statistical significance was set at P = 0.05.

Frequencies and percentages were used to describe raw data. Proportional adverse event mortality (per 1000 deaths) was calculated as adverse event deaths divided by total deaths × 1000. First, proportional mortality was calculated independently for each combination of year, sex, ethnicity, age, and urbanization level. Then, standardized mortalities of urbanization levels were calculated following 2010 distibution as the reference. Temporal patterns among urbanization levels were illustrated by mortality plots.

A two-level linear mixed model was constructed, using standardized proportional mortality (adverse event deaths per 1000 inpatient deaths) as the outcome, and urbanization level, year, and urbanization level*year as predictor terms. The most urban category (i.e., large metropolitan) was used as reference. Years were considered to be nested within urbanization levels. The year variable was mean-centered. Beta coefficients, 95% confidence intervals (CI), and P values were obtained from the data output.

## 3 Results

Of the 8 071 907 certified inpatient deaths during the study period, 21 444 (0.27%) were primarily attributed to medical adverse events. Most adverse event deaths were procedure-related (83.3%). Table 1 presents an annual summary of inpatient deaths.

**Table 1:** Annual summary of total inpatient deaths, adverse event deaths, and decedents’ demographics. Values are frequencies and percentages unless otherwise indicated.

	2010		2011		2012		2013		2014		2015		2016		2017		2018		2019	
Total inpatient deaths	814 404		811 114		792 223		793 903		793 403		808 260		807 402		818 522		819 467		813 209	
All adverse events^a^	1698	2.1	1690	2.1	1675	2.1	1627	2.0	1658	2.1	1716	2.1	2072	2.6	2928	3.6	2981	3.6	3399	4.2
Procedure-related events^a^	1331	1.6	1354	1.7	1351	1.7	1297	1.6	1353	1.7	1385	1.7	1730	2.1	2513	3.1	2573	3.1	2975	3.7
Other adverse events^a^	367	0.5	336	0.4	324	0.4	330	0.4	305	0.4	331	0.4	342	0.4	415	0.5	408	0.5	424	0.5

Sex																				
Male	419 555	51.5	418 962	51.7	410 948	51.9	413 925	52.1	417 001	52.6	425 381	52.6	427 638	53.0	434 164	53.0	436 789	53.3	434 701	53.5
Female	394 849	48.5	392 152	48.3	381 275	48.1	379 978	47.9	376 402	47.4	382 879	47.4	379 764	47.0	384 358	47.0	382 678	46.7	378 508	46.5

Ethnicity																				
White	669 256	82.2	666 993	82.2	649 326	82.0	648 616	81.7	647 152	81.6	657 716	81.4	652 972	80.9	661 697	80.8	659 211	80.4	653 534	80.4
Other	145 148	17.8	144 121	17.8	142 897	18.0	145 287	18.3	146 251	18.4	150 544	18.6	154 430	19.1	156 825	19.2	160 256	19.6	159 675	19.6

Age (years)																				
0 to 29	33 570	4.1	33 290	4.1	32 774	4.1	32 542	4.1	32 360	4.1	33 034	4.1	33 960	4.2	33 115	4.0	32 002	3.9	31 032	3.8
30 to 59	140 390	17.2	140 791	17.4	137 616	17.4	138 282	17.4	140 268	17.7	139 392	17.2	142 293	17.6	140 953	17.2	140 118	17.1	138 115	17.0
60+	640 444	78.6	637 033	78.5	621 833	78.5	623 079	78.5	620 775	78.2	635 834	78.7	631 149	78.2	644 454	78.7	647 347	79.0	644 062	79.2

Urbanization level																				
Large metropolitan	412 084	50.6	411 944	50.8	400 761	50.6	400 608	50.5	399 725	50.4	408 124	50.5	409 108	50.7	416 012	50.8	416 894	50.9	414 539	51.0
Medium or small metropolitan	244 019	30.0	242 725	29.9	239 087	30.2	240 716	30.3	242 333	30.5	247 046	30.6	247 325	30.6	250 984	30.7	251 527	30.7	249 342	30.7
Nonmetropolitan	158 301	19.4	156 445	19.3	152 375	19.2	152 579	19.2	151 345	19.1	153 090	18.9	150 969	18.7	151 526	18.5	151 046	18.4	149 328	18.4

a Values are frequencies and proportions relative to 1000 inpatient deaths.

**Fig. 1:**
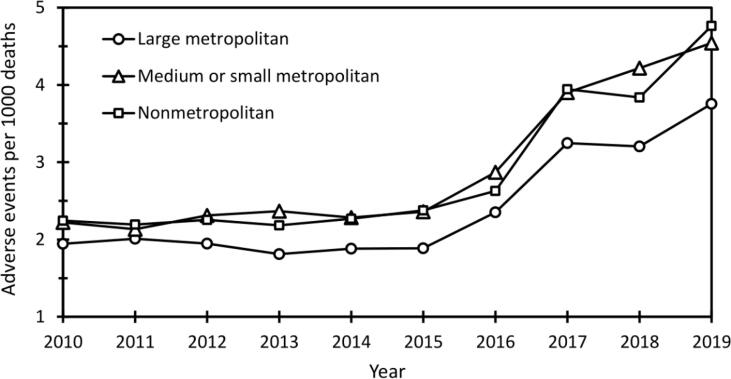
All adverse event deaths per 1000 inpatient deaths (standardized for sex, ethnicity, and age) in urbanization categories over the period 2010–2019.

Fig. 1 demonstrates fatal adverse events in urbanization categories across the study period; the corresponding linear mixed model is presented in Table 2. The analysis revealed urban–rural gradients in proportional adverse event mortality. Decedents from large metropolitans had the lowest numbers of fatal adverse events relative to total inpatient deaths throughout the study period, ranging between 1.8 and 3.8 per 1000 deaths. Decedents from medium or small metropolitans and nonmetropolitans had on average 0.5 units higher adverse event rates (p < 0.001), ranging between 2.1 and 4.8 per 1000 deaths. Moreover, the urban–rural gradients showed an increasing trend towards the end of the study period, as the difference between decedents from large metropolitans and those from more rural settings was found to increase at a rate of approximately 0.1 units per year (p < 0.001). There were no statistically significant differences between decedents from medium or small metropolitans and nonmetropolitans.

**Table 2:** Linear mixed model with the number of all adverse event deaths per 1000 inpatient deaths (standardized for sex, ethnicity, and age) as outcome.

Predictor	Beta coefficient	95% confidence interval	P value
Intercept	**2.40**	**2.15; 2.66**	**< 0.001**
Year	**0.20**	**0.11; 0.29**	**< 0.001**
Urbanization level			
Large metropolitan	Reference		
Medium or small metropolitan	**0.52**	**0.43; 0.60**	**< 0.001**
Nonmetropolitan	**0.47**	**0.39; 0.56**	**< 0.001**
Urbanization level*year			
Large metropolitan	Reference		
Medium or small metropolitan	**0.07**	**0.05; 0.10**	**< 0.001**
Nonmetropolitan	**0.07**	**0.04; 0.10**	**< 0.001**

## 4 Discussion

This study aimed to compare the distribution of deaths due to medical adverse events across urbanization levels among US hospital inpatients over the period 2010–2019. The analysis revealed urban–rural gradients in proportional adverse event mortality. Decedents who resided in medium or small metropolitans and nonmetropolitans had approximately 0.5 units higher rate of adverse events per 1000 deaths (corresponding to a relative differece of 20%) when compared to decedents who resided in large metropolitans. Moreover, the urban–rural gradients showed an increasing trend towards the end of the study period, as the difference was found to increase at a rate of approximately 0.1 units per year (3%). There was no statistically significant difference between decedents from medium or small metropolitans and nonmetropolitans. As such, the a priori hypothesis was partially confirmed.

While ca. 40% of the US population reside in predominantly urban areas, another 40% reside in predominantly rural areas (Brezzi et al., 2011). It is thus clear that the present findings, obtained from a national dataset extending over a period of 10 years, are relevant for a broad population base. Unlike most previous studies (Atmore et al., 2021, Coburn et al., 2004), this study focused on deaths primarily attributed to adverse events, as they arguably constitute the most severe outcome in the patient safety context. Adverse event deaths were studied in relation to total inpatient deaths to account for trends in overall mortality. A comprehensive assessment of patient safety on a national level is challenging, with varying results depending on the dataset (Sunshine et al., 2019). The present study utilized the official NCHS mortality data.

Understanding how urbanization level relates to an inpatient’s risk of sustaining medical adverse events will help highlight geographic areas at highest risk and direct preventive efforts accordingly. In this dataset, adverse event deaths were least likely in the most urban category, and more common in the two remaining (more rural) categories. As such, the findings were generally in contrast to previous reports (Atmore et al., 2021, Coburn et al., 2004, Vartak et al., 2010) but in line with the present hypothesis. With regard to previous reports, the differing geographical coverages, time periods, definitions of urbanity and rurality, and adverse event outcomes should be acknowledged as potential sources of discrepancy. Of note is also the fact that the present analysis addressed sex-, ethnicity-, and age-standardized proportional mortality, as true population denominators and other adjustments were not available.

Although the present data are heavily limited in terms of elucidating any underlying factors, speculative explanations for the findings may be offered. In the US, rural areas are prone to physician shortages, socioeconomic deprivation, and have a higher rate of individuals with no health insurance (Gong et al., 2019), and these aspects may explain the higher rate of adverse event deaths. Individuals residing in rural communities may also not seek care as actively as those in urban areas. Narrower availability of medical services, frequent need for patient transfers, or treatment delays due to, e.g., longer distances between medical units may correspondingly account for the higher rate of adverse events. Factors generally associated with a higher risk of adverse events, such as care-seeking delay, severity of condition at presentation, required treatment, and previous complications, may also prove important. Temporal shifts in these factors may also account for the increase in gradients over time. However, the present data did not include records as to where the fatal adverse events occurred. Future studies are warranted to characterize the underlying factors in detail. The National Inpatient Survey and Global Burden of Diseases databases, for example, may prove fruitful in further analyses.

Large metropolitans set aside, the remaining categories (medium or small metropolitans and nonmetropolitans) had a similar rate of adverse event deaths, with no statistically significant difference between the two. This is relatively surprising, as a somewhat linear pattern in effect size was expected between the three categories. In this sense, the findings were not in line with the hypothesis, and unfortunately, more detailed analyses are outside the reach of the dataset. However, the present findings do imply that the potential explanatory factors lie on – and should be primarily sought from – the borderline between large metropolitans and medium or small metropolitans. It may be beneficial to consider this finding while planning future approaches to the topic.

The main strengths of this study were an official data source, national coverage of all certified US deaths, and a decade-long timespan. Moreover, urbanization level was assigned to each death in accordance with an official classification scheme. Importantly, the dataset is publicly available for confirmatory and subsequent analyses. The main limitations were the lack of background data on the deaths and decedents, and low numbers of some adverse event subtypes. Most importantly, the dataset lacked information as to where the care was received and where the fatal adverse event occurred. Although mortality was standardized for sex, ethnicity, and age, there may well be residual confounding. Risk factors of adverse events (e.g., care-seeking delay, severity of condition, required procedures, previous complications) were not accounted for. In this study, NCHS mortality data were taken as ‘face value’. However, there have been concerns whether adverse events are efficiently and accurately captured in mortality datasets (Makary and Daniel, 2016, Oura, 2021). The data appear prone to diagnostic and coding errors; the potential errors in cause-of-death coding may vary by region depending on, e.g., whether death certificates are filled by medical staff or not. While this analysis focused on deaths primarily attributed to adverse events, future studies are welcomed to address adverse events as contributory causes of death.

## 5 Conclusion

This retrospective register-based study exploited US nationwide cause-of-death data from 2010 to 2019, aiming to compare the distribution of deaths due to medical adverse events across urbanization levels among hospital inpatients. Decedents who resided in medium or small metropolitans and nonmetropolitans had approximately 0.5 units higher rate of adverse events per 1000 deaths (corresponding to a relative differece of 20%) when compared to decedents who resided in large metropolitans. Moreover, the urban–rural gradients showed an increasing trend towards the end of the study period, as the difference was found to increase at a rate of approximately 0.1 units per year (3%). There was no statistically significant difference between decedents from medium or small metropolitans and nonmetropolitans. The present findings highlight differences in adverse event deaths between geographic areas, providing a basis for targeted preventive efforts. Future studies are invited to elucidate the underlying phenomena, bearing in mind that the explanatory factors may exist on the borderline between large metropolitans and medium or small metropolitans.

## Funding

Open access funded by Helsinki University Library.

## CRediT authorship contribution statement

**Petteri Oura:** Conceptualization, Methodology, Formal analysis, Data curation, Writing – original draft, Writing – review & editing.

## Declaration of Competing Interest

The author declares that he has no known competing financial interests or personal relationships that could have appeared to influence the work reported in this paper.
